# Zearalenone Affect the Intestinal Villi Associated with the Distribution and the Expression of Ghrelin and Proliferating Cell Nuclear Antigen in Weaned Gilts

**DOI:** 10.3390/toxins13100736

**Published:** 2021-10-19

**Authors:** Quanwei Zhang, Libo Huang, Bo Leng, Yang Li, Ning Jiao, Shuzhen Jiang, Weiren Yang, Xuejun Yuan

**Affiliations:** 1 College of Animal Sciences and Technology, Shandong Agricultural University, Tai’an City 271018, China; sdndzqw@163.com (Q.Z.); huanglibo@sdau.edu.cn (L.H.); lengbo666@163.com (B.L.); liyang_cc@yeah.net (Y.L.); jiaoning@sdau.edu.cn (N.J.); shuzhen305@163.com (S.J.); 2 College of Life Sciences, Shandong Agricultural University, Tai’an City 271018, China

**Keywords:** zearalenone, weaned gilt, intestinal morphology, ghrelin, PCNA

## Abstract

This study explored and investigated how zearalenone (ZEA) affects the morphology of small intestine and the distribution and expression of ghrelin and proliferating cell nuclear antigen (PCNA) in the small intestine of weaned gilts. A total of 20 weaned gilts (42-day-old, D × L × Y, weighing 12.84 ± 0.26 kg) were divided into the control and ZEA groups (ZEA at 1.04 mg/kg in diet) in a 35-d study. Histological observations of the small intestines revealed that villus injuries of the duodenum, jejunum and ileum, such as atrophy, retardation and branching dysfunction, were observed in the ZEA treatment. The villi branch of the ileum in the ZEA group was obviously decreased compared to that of the ileum, jejunum and duodenum, and the number of lymphoid nodules of the ileum was increased. Additionally, the effect of ZEA (1.04 mg/kg) was decreased by the immunoreactivity and distribution of ghrelin and PCNA in the duodenal and jejunal mucosal epithelial cells. Interestingly, ZEA increased the immunoreactivity of ghrelin in the ileal mucosal epithelial cells and decreased the immunoreactivity expression of PCNA in the gland epithelium of the small intestine. In conclusion, ZEA (1.04 mg/kg) had adverse effects on the development and the absorptive capacity of the villi of the intestines; yet, the small intestine could resist or ameliorate the adverse effects of ZEA by changing the autocrine of ghrelin in intestinal epithelial cells.

## 1. Introduction

*Fusarium*, a kind of fungi, is widely distributed in nature and is common in North America, Asia and Europe with mild climates [1]. Mycotoxins are produced by *Fusarium*, which is a major and serious threat to animal and human health, as well as livestock production [2,3,4]. In terms of animal health and productivity, the most important mycotoxins were trichothecenes, zearalenone (ZEA), deoxynivalenol and fumonisins B_1_ [5,6]. The gastrointestinal tract is one of the most sensitive tissues to these mycotoxins [1]. Studies have shown that mycotoxins can damage animal intestines through impairing the reduction–oxidation reaction balance of the body, affecting the digestive tract function and causing intestinal villus atrophy and an inflammatory response in the intestinal epithelial cells of piglets [3,7,8]. Some in vitro studies have shown that food contaminated by ZEA and ZEA metabolites can affect the synthesis of porcine cytokines and the structural integrity of the intestinal epithelium [9,10]. The results of the differential gene expression and microarrays showed that there were 190 differently expressed genes in isolated IPEC-1 (porcine epithelial cells) treated with ZEA, of which 70% were upregulated [11]. A low dose (40-μg/kg BW) of ZEA did not change the mucosa thickness, villus length and villus-to-crypt of the duodenum [12]. As far as we know, there are few published in vivo studies available on the evaluation of the impacts of ZEA exposure on the intestinal structure and function of gilts.

The small intestine is a critical place where nutrients are absorbed. Intestinal epithelial cells are the first important and physical barrier to avoid the gastrointestinal absorption of toxins [13], as well as the first target of toxins [14,15]. Ghrelin is a pleiotropic hormone, which can promote growth hormone secretion [16], increase food intake [17], regulate the energy metabolism and intracellular homeostasis [18,19] and is even involved in the immune regulation of the intestinal mucosa [20]. Blood ghrelin mainly comes from the gastrointestinal tract; a small amount of circulating ghrelin comes from the immune system, kidney, pancreas, testis, ovary and placenta [21]. Research in pigs showed that ghrelin also influenced or regulated their growth and development [22]. The results from Willemen illustrated that the amount of expressing active ghrelin in gastric cells in the normal weight neonates was higher compared with the small-for-gestational age piglets [23]. A previous study reported that a diet containing ZEA could affect the ovarian histology and follicular development by affecting the expression of PCNA and ghrelin in the ovaries [24] and increasing the PCNA expression of granulosa cells and then accelerate the changes in ovarian histology and the development of ovaries in the weaned gilts [24]. However, information was limited on the effects of a diet containing ZEA exposure on the expression and distribution of ghrelin and PCNA in the small intestine of weaned gilts.

The purpose of this research was to evaluate the impacts of ZEA in the diet on the histological structure and the mRNA and protein expressions of ghrelin and PCNA in the small intestine of weaned gilts. The results will be helpful to evaluate the impacts of a 1.04-mg/kg dose of ZEA in a diet on the subsequent damage and the digestive capacity of the small intestines in weaned gilts.

## 2. Results

### 2.1. Serum ZEA, α-ZOL, and β-ZOL

The results showed that the serum α-ZOL (α-zearalenol), β-ZOL (β-zearalenol) and ZEA contents in the ZEA group were higher than those in the control group (Table 1, *p* < 0.05). These confirmed that ZEA could be absorbed and partially degraded into β-ZOL and α-ZOL by the liver.

### 2.2. Morphological Structure and Measurement

**Figure 1:**
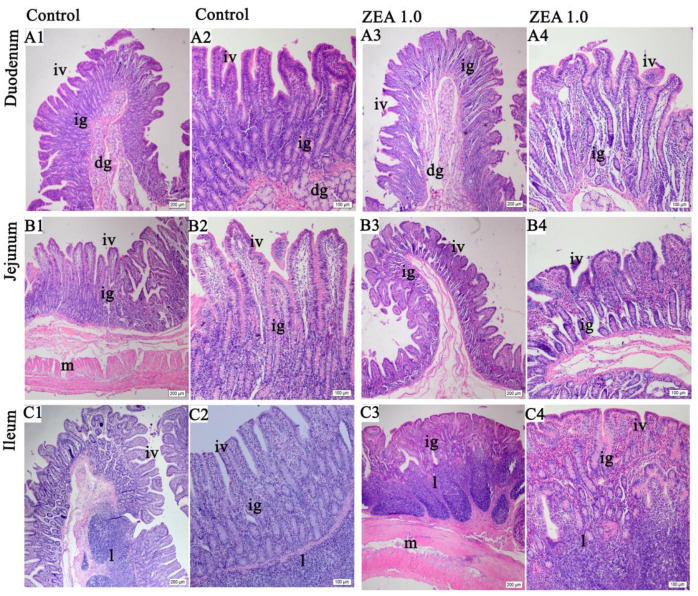
Representative hematoxylin and eosin staining images of the small intestine in weaned gilts (*n* = 6). (**A1**,**A2**,**B1**,**B2**,**C1**,**C2**) were the control treatment, and (**A3**,**A4**,**B3,B4**,**C3**,**C4**) were the ZEA1.0 treatment. Scale bars were 200 µm for (**A1**,**A3**,**B1**,**B3**,**C1**,**C3**) and 100 μm for (**A2**,**A4**,**B2**,**B4**,**C2**,**C4**), respectively. ig represented intestinal gland, iv represented intestinal villus, g represented duodenal gland, l represented lymphoid nodule and m represented musculari.

Compared with the gilts in the control treatment, the gilts in the ZEA treatment showed intestinal villus injuries, such as shortening, retardation and branching dysfunction, and more obvious morphological changes of the villus branch in the jejunum and ileum (Figure 1B1–C4). The duodenal villi in the ZEA treatment displayed a finger-like structure (Figure 1A3,A4), but the duodenal villus length of the gilts in the control treatment was longer than those of the ZEA treatment (Figure 1A1–A4). The jejunal villi of the gilts in ZEA treatment group were stubby and leaf-like (Figure 1B3,B4), but the jejunal villi of the gilts in the control treatment were finger-like (Figure 1B1,B2). The branches of the ileal villi in the ZEA treatment were obviously incomplete (Figure 1C3,C4), and the number and the area of the ileal submucosal lymph nodes were increased.

The parameters of the morphometric analysis of the small intestine in the control and ZEA treatment are listed in Table 2. The results showed that the villi lengths-to-crypt depth and villus length of ZEA exposure were significantly reduced (*p* < 0.05), and the crypt depth of the ZEA treatment became thick (*p* < 0.05) compared to the control treatment in the intestinal three segments.

### 2.3. The Ghrelin Immunoreactive Cells Distribution

**Figure 2:**
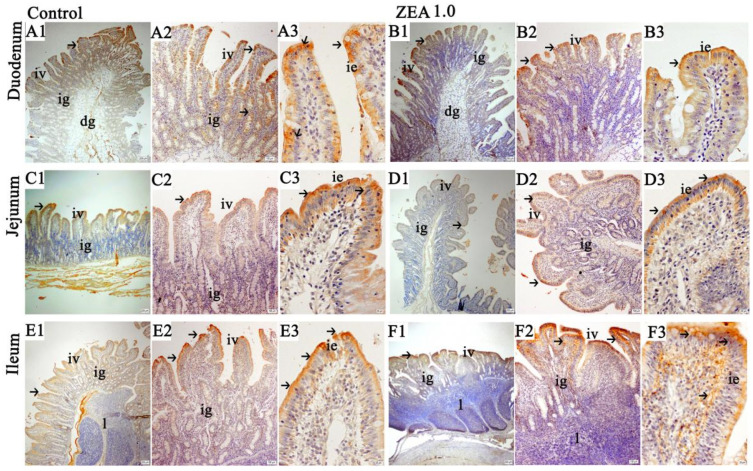
Representative distribution of ghrelin immuno-positive cells in the small intestine of weaned gilts (*n* = 6). (**A1**–**A3**,**C1**–**C3**,**E1**–**E3**) were the control, and (**B1**–**B3**,**D1**–**D3**,**F1**–**F3**) were the ZEA1.0 treatment. Scale bars of (**A1**,**B1**,**C1**,**D1**,**E1**,**F1**) were 200 μm, of (**A2**,**B2**,**C2**,**D2**,**E2**,**F2**) were 100 μm and of (**A3**,**B3**,**C3**,**D3**,**E3**,**F3**) were 20 μm. ig represented the intestinal glands, iv represented the intestinal villus, dg represented the duodenal gland, ie represented the intestinal villus epithelium, l represented the lymphoid nodule and m represented the muscularis.

**Figure 3:**
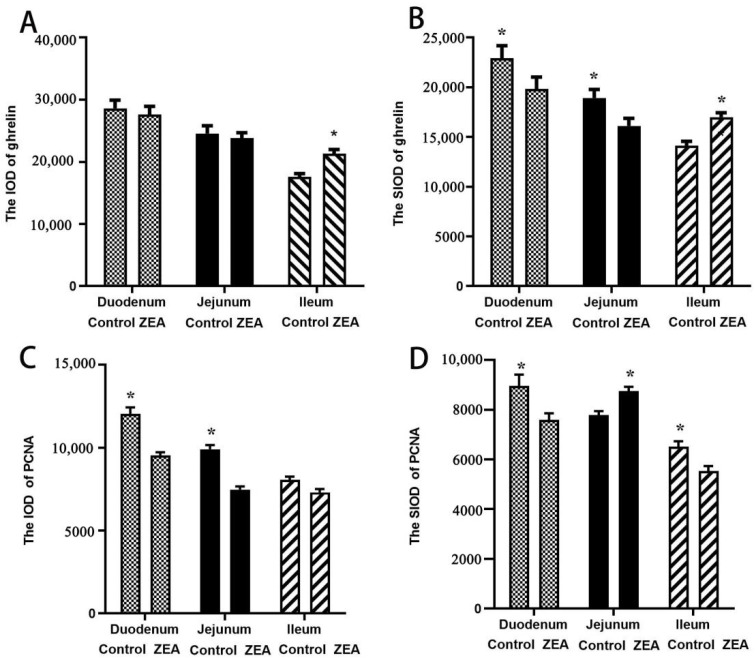
The IOD and SIOD of ghrelin and PCNA of the small intestine in weaned gilts (*n* = 6). * Means were significantly different (*p* < 0.05). (**A**) The IOD of ghrelin, (**B**) the SIOD of ghrelin, (**C**) the IOD of PCNA and (**D**) the SIOD of PCNA.

The distribution and expression of ghrelin in the small intestine were presented in Figure 2. The immunohistochemical results showed that ghrelin-positive substances were distributed in the cytoplasm of the villi and glandular epithelium. The ghrelin immunoreactivity was stronger in the villi mucosal epithelium adjacent to the intestinal lumen at the apical surface of the folds, but it was gradually reduced and weakened in epithelial cells on both sides and near the base. The ghrelin localization pattern of the duodenum (Figure 2A1–B3) and jejunum (Figure 2C1–D3) in the control treatment (Figure 2A1–A3,C1–C3) was essentially the same as that in the ZEA treatment (Figure 2B1–B3,D1–D3), but in the ileal mucosal epithelial cells, ZEA increased the ghrelin immunoreactivity compared to the control (Figure 2E1–F3). The results of a single villus’ integrated optic density (SIOD) of duodenal and jejunal ghrelin in weaned gilts were consistent with the above results of the immunochemical analysis. The SIOD of the ZEA group were obviously decreased compared to those in the control treatment (*p* < 0.05, Figure 3B). However, the IOD and SIOD of the ileal ghrelin in the ZEA group were increased significantly compared to those in the control group (Figure 3A,B, *p* < 0.05).

### 2.4. The Distribution of PCNA Immunoreactive Cells

**Figure 4:**
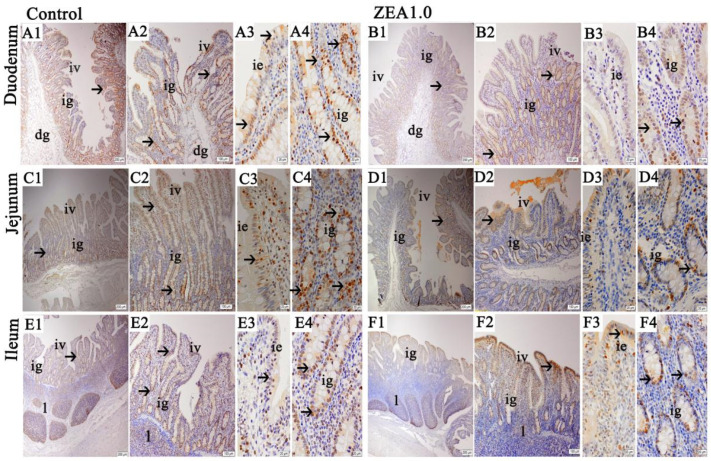
Representative distribution of PCNA immuno-positive cells of the small intestine in weaned gilts. (**A1**–**A4**,**C1**–**C4**,**E1**–**E4**) were the control treatment, and (**B1**–**B4**,**D1**–**D4**,**F1**–**F4**) were the ZEA1.0 treatment. Scale bars were 200 μm for (**A1**,**B1**,**C1**,**D1**,**E1**,**F1**), 100 μm for (**A2**,**B2**,**C2**,**D2**,**E2**,**F2**) and 20 μm for (**A3**,**A4**,**B3**,**B4**,**C3**,**C4**,**D3**,**D4**,**E3**,**E4**,**F3**,**F4**). ig represented the intestinal gland, iv represented the intestinal villus, dg represented the duodenal gland, ie represented the intestinal villus epithelium, l represented the lymphoid nodule and m represented the muscularis.

The expression and distribution of PCNA in the small intestines of gilts were detected (Figure 4). The immunohistochemical results showed that PCNA-positive substances were distributed in small intestinal villi and intestinal glands. The PCNA immunoreactivity was stronger in villus epithelial cells, especially at the bottom of the small intestinal villi, but weakened gradually in the villus epithelial cells of both sides and at the top of the small intestinal villi. The location pattern of PCNA in the duodenum (Figure 4A1–A4), jejunum (Figure 4C1–C4) and ileum (Figure 4E1–E4) of the control treatment were basically the same as those in the ZEA treatment (Figure 4B1–B4,D1–D4,F1–F4). The results of the ZEA treatment showed that the PCNA immunoreactivity of the intestinal gland cells in the duodenum, jejunum, ileum (Figure 4A4,B4,C4,D4,E4,F4) and villus epithelial cells at the base of the small intestinal villi (Figure 4A3,B3,C3,D3,E3,F3) were significantly weaker than those of the control treatment. The results of the IOD of duodenal and jejunal PCNA and SIOD in the duodenal, jejunal and ileal PCNA revealed that those of the ZEA group were significantly lower than those of the control group (Figure 3C,D, *p* < 0.05).

### 2.5. The mRNA and Protein Relative Expressions of Ghrelin and PCNA

**Figure 5:**
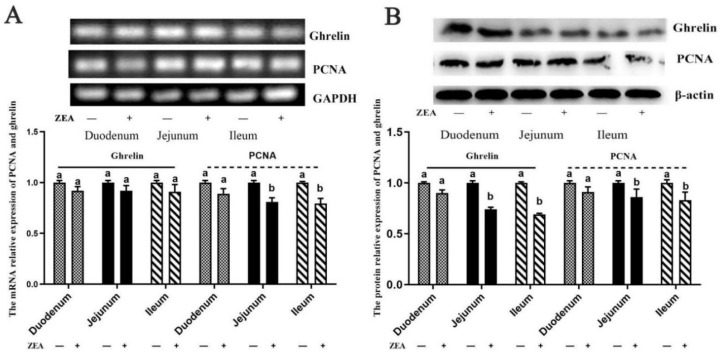
The protein and mRNA relative expressions of ghrelin and PCNA in the small intestine (duodenum, jejunum and ileum) in weaned gilts (*n* = 4). (**A**) The mRNA relative expressions of ghrelin and PCNA, and (**B**) the protein expressions of ghrelin and PCNA. ^a,b^ Means differ significantly (*p* < 0.05).

The mRNA relative expression of ghrelin was consistent with the immunohistochemistry analysis above. Compared to the control treatment, the decreased mRNA relative expression of the ileal and jejunal PCNA (Figure 5A, *p* < 0.05) were also observed in the ZEA treatment, but there were no obvious differences in the duodenal ghrelin and PCNA mRNA relative expressions that were observed (*p* > 0.05) between the control and ZEA treatments (Figure 5A).

The results from the protein expressions of ghrelin and PCNA in the jejunum and ileum showed that the ZEA-treated gilts were significantly decreased compared to the control gilts (*p* < 0.05, Figure 5B).

## 3. Discussion

The meaningful findings of this study were that ZEA might damage the intestinal structure by changing the expression of ghrelin and PCNA. It was found that prepubertal pigs might be very sensitive animals to ZEA toxicity [25]. The estrogenic properties of ZEA have been reported extensively in the literature. Yang et al. [26] and Zinedine et al. [27] found that ZEA caused a high estrogen syndrome in animals, leading to reproductive disorders and infertility, ovarian and uterine dilation and decreased pregnancy rates in pigs and cattle [26,27]. Importantly, the small intestine acts as the first line of defense for ZEA, which is mainly absorbed into the intestinal tract and can cause intestinal damage. Liu et al. [28] reported that HSP70 expression and MDA content in the small intestine (duodenum, jejunum and ileum) were increased in weaned gilts fed a 1.04-mg/kg ZEA diet. The meaningful findings of this study were that ZEA might damage the intestinal structure by changing the expression of ghrelin and PCNA.

### 3.1. Morphological Structure of Small Intestine and Serum ZEA, α-ZOL and β-ZOL

Studies have shown that the small intestine is the key and main part of the absorption of most nutrients. Therefore, the changes in the structure and function of the small intestinal mucosa are closely related to the digestion and absorption of nutrients. Moreover, the villi, finger-like protrusions of the small intestine expand the surface area of the mucosa and are arranged within the intestinal mucosa epithelial cell layer facing the lumen to form a protective barrier to protect the body from direct contact with microorganisms and food antigens [29]. ZEA can cause intestinal damage, which would lead to the impairment of the intestinal absorption capacity and barrier function [30,31]. Intestinal morphology changes caused by mycotoxins (deoxynivalenol and ZEA) affected the defense mechanisms of the large intestine, and the number of plasma cells and lymphocytes also increased [8,32,33,34,35]. The report confirmed that immature gilts were administered ZEA (40-μg/kg BW) orally one week, and the number of lymphocytes and goblet cells in the intestinal villus mucosal epithelium obviously increased [36,37,38]. Especially the results of the ileum and jejunum in this study, ZEA exposure damaged the structure of the small intestine by shortening the villi and destroying the branching function of the ileum and jejunum and the decreased villus length of the duodenum, jejunum and ileum treated with ZEA. In the current study, it was hinted that 1.04-mg/kg ZEA resulted in the reduction of the functional mucosal epithelial surface area; the nutrient absorption capacity might decrease in a short time period. However, Liu et al. [28] found no significant difference in the ADFI, ADG or feed efficiency (ADG/ADFI) in gilts when the gilts were treated with ZEA at 1.04 mg/kg in a diet) for 35 days. Therefore, the grow performance index was not a sensitive index of short-term intestinal injury, the morphological structure and the index of the length of the small intestine villi, and the depth of the crypts was more accurate for evaluating the effect of ZEA on the intestinal morphology. This result also prompted that ZEA was mainly absorbed through small intestinal cells; after enterohepatic circulation, ZEA was degraded into β-ZOL and α-ZOL by the liver and then combined with glucuronic acid. Although ZEA and its metabolites were finally excreted through feces, urine or milk, ZEA and its metabolites would still accumulate in target organs in animals and endanger animal health [39]. The higher contents of α-ZOL, β-ZOL and ZEA in the serum of the ZEA treatment indicated that dietary 1.04-mg/kg ZEA could be absorbed by intestinal epithelial cells and degraded in the liver and act on reproductive organs and other target organs through blood circulation.

### 3.2. The Distribution and Expression of Ghrelin

Ghrelin, a brain–gut peptide consisting of 26 amino acids, has attracted widespread attention since it was discovered and plays a protective role in animal gastrointestinal injury [40]. Ghrelin is most densely distributed in the gastric and intestine mucosa of various vertebrates (mammals and nonmammals) [41,42]. The intestinal recess and villous cells greatly reduce the number of ghrelin with the backward extension of the digestive tract [43]. Reports were confirmed that the main secretory segment of ghrelin was the duodenum [44]; the number of ghrelin results in this research showed that the secretion of ghrelin in the duodenal and jejunal mucosa of the ZEA treatment decreased, which might be the normal physiological defense response of intestinal epithelial cells to a reduced ZEA absorption. The function of the jejunum is to digest and absorb food. Moreover, ghrelin was found to affect the food intake, endocrine regulation of intestinal emptying and motility in rats/mice [45], and in human metabolic activities, it has been reported that ghrelin is associated with intestinal mucosa [21,46,47]. The domestic research showed that ghrelin acted as a gastrointestinal hormone to stimulate the appetite, increase the feed intake and regulate the energy balance [48,49]. However, the high expression of ghrelin (IOD and SIOD) in the ileum of the control was lower than those of the ZEA treatment, which we believed that might be related the immune function of the ileum to resist the toxicity of ZEA. Our results prompted us to relate the results of the Keap1–Nrf2 signaling pathway, which was likely activated by ZEA in the ileum [50]. ZEA may have exerted influence on the hormone/signal molecule secretion rule of the small intestine. Previous studies showed that ghrelin is potentially therapeutic for mucosal injuries and intestinal permeability [51,52]. Hatoya et al. [53] found that higher estrogen receptor levels caused lower ghrelin mRNA levels. This study hinted that ZEA could cause a decrease in the secretion of ghrelin in the duodenal and jejunal mucosa, which might be the normal physiological defense response of intestinal epithelial cells to a reduced ZEA absorption. However, its molecular mechanism needs to be further confirmed and further studied, because the effects of ghrelin on ZEA induced the toxicity of the ileum in vitro.

### 3.3. The Distribution and Expression of PCNA

It had been confirmed that the renewal and proliferation of intestinal villi epithelial cells were migrated from the crypt to the top of the villi and that the continuous proliferation and differentiation of intestinal stem cells in the intestinal crypts could achieve a renewal of the intestinal epithelium, so the proliferation rate of the epithelial cells and the rate of apoptosis and shedding of the epithelial cells were closely related to the growth of the intestinal villi [54]. The PCNA exists in normal proliferative cells and tumor cells, PCNA was also a major endogenous marker for testing the cell proliferation ability [55]. In addition, PCNA also plays an important role in the post-traumatic repair of many intestinal diseases. Liu et al. [56] confirmed that ZEA at 1.04 mg/kg in a diet exerted immunotoxicity and cytotoxicity through inducing oxidative stress, and then led to apoptosis and DNA oxidative damage. However, there were reports that an alternative pathway of DNA damage was closely connected to the monoubiquitination of PCNA [57,58]. In the current study, we also found that ZEA decreased the expression of PCNA in the villus epithelium and increased the expression of PCNA in the intestinal gland, which was consistent with ZEA destroying the branching and damaging the structure of the small intestine. The increased expression of PCNA in small intestinal glands showed the importance of PCNA, which was related to the self-repair mechanism of the small intestine. In addition, the results of the PCNA in the present study also indicated the influence and damage of 1.04-mg/kg ZEA to the gilts’ small intestines, but their bodies still had the ability to be repaired; however, we suspected that the repair ability of the small intestine in the resistance to ZEA injury and the effect of ZEA on PCNA expression might occur via destroying the proliferation ability of small intestinal stem cells, which drive the renewal and rebirth of the intestinal epithelium every 2 to 3 days [59]. Therefore, further studies were needed to study the damaging effects of ZEA on intestinal stem cells and clarify the potential toxic effect of ZEA.

## 4. Conclusions

In conclusion, under the experimental conditions, we detected the influence of 1.04-mg/kg ZEA on the morphological structure of the small intestine in weaned gilts and the expression and distribution of ghrelin and PCNA, which act as important markers of intestinal development and functionality. The results also suggested that ZEA might have adverse effects on the health and growth of gilts at a later stage. More in vivo and in vitro studies are needed to confirm the relationship among the nutrient absorption, growth performance of pigs and intestinal injury caused by ZEA.

## 5. Materials and Methods

### 5.1. Ethics Statement, Experimental Design, Animals and Treatments

In this experiment, the gilts were fed in accordance with the Guide for the Care and Use of Laboratory Animals, which were approved by the Committee on the Ethics of Shandong Agricultural University (ID: S20180058, date of approval 10 December 2019).

Twenty healthy D × L × Y weaned gilts (Duroc × Landrace × Yorkshire) aged 42 ± 2 d (average weight 12.84 ± 0.26 kg) were placed in individual 0.48 m^2^ cages. All gilts were fed ad libitum and had free access to water, the relative humidity of the room was approximately 65% and the temperature maintained between 26 and 28 °C during the whole experimental period. Gilts were randomly divided into two treatments (10 gilts per treatment) to receive a basal diet or a 1.0-mg/kg dose ZEA diet (basal diet added with 1.0-mg/kg ZEA). The zearalenone dosage used in this current study was due to previous investigations and the recent literature [60,61,62,63], according to Liu et al. [28], for 35 d following a 7-d adaptation.

The basal diet (Table 3) was selected to meet or exceed the piglets’ nutrient requirements recommended by the NRC 2012 (National Research Council). The ZEA diet was prepared according to the studies by Dai et al. [24] and Liu et al. [28]. The two diets were produced in the same batch and stored in prior to feeding. The nutrient composition of the experimental diets was analyzed according to the method in which were described by the 2012 AOAC (Association of Official Analytical Chemists). According to Zhou et al. [64] and Liu et al. [28], the diet sample was collected before and at the end of the experiment; then, we determined the toxin contents in the diets (Table 4) The LC-MS method high-performance liquid chromatography (HPLC) tandem mass spectrometry (MS) with the fluorescence detection method, affinity column chromatography method and the external standard method were used to quantify FUM (fumonisin) and DON (deoxynivalenol) and AFL (aflatoxin). Using the LC method in conjunction with the fluorescence detection method, affinity column chromatography method, and the external standard method quantified ZEA. Its minimum detection concentration of the toxins was 0.05 mg/kg for DON, 1.0 μg/kg for AFL, 0.1 mg/kg for FUM and 0.01 mg/kg for ZEA.

The measured results of the toxins showed that the concentrations of the aflatoxins (DON, AFL, FUM and ZEA) in the basal diets were 0.41 mg/kg, 1.62 µg/kg, 0.28 mg/kg and 0.14 mg/kg and were 0.41 mg/kg, 1.59 µg/kg, 0.28 mg/kg and 1.04 mg/kg in the ZEA diet.

### 5.2. Sample Collection and Preparation

After the experiment was completed, all gilts were fasted for 10–12 h and then blood and separated serum were collected. The serum samples of the gilts were taken and kept at 20 °C for the ZEA content analysis. Collected samples (2.0–2.5 cm in length, approximately) from the middle of the duodenum, jejunum and ileum were immediately extracted after evisceration. One sample was stored in a RNase-free frozen tube, then placed and immersed in the liquid nitrogen immediately and kept at the ultra-low temperature of −80 °C in a refrigerator for the subsequent analysis of the expression of mRNA and protein in the small intestine; another sample was fixed in Bouin’s solution for the following histological and immunohistochemical examinations.

### 5.3. The Concentrations of ZEA, β-ZOL and α-ZOL during Serum Detection

The serum concentrations of ZEA, α-ZOL and β-ZOL were analyzed by the Institute of Quality Standards and Detection Technology of Chinese Academy of Agricultural Sciences according to Liu et al. [28]. A LC-MS/MS analysis was performed using an Agilent 1200 liquid chromatograph (Agilent Technologies, Palo Alto, CA, USA) coupled to a 3200 QTrap^®^ mass spectrometry system (Applied Biosystems, Foster City, CA, USA) equipped with a Turbo electrospray ionization (ESI) interface.

### 5.4. Small Intestine Histology Examination

The fixed small intestinal tissues were treated with a gradient series of ethanol and xylene solutions and then embedded conventionally in the paraffin wax. The embedded tissues were sliced to 5-μm-thick sections and stained with the dye of the hematoxylin and eosin (H&E staining method) to observe the small intestinal tissue structure under light microscopy. Morphometric analyses were performed using microscope analyses software (Olympus BX41, Tokyo, Japan), and the parameters, including the crypt depth, villus length and VL/CD (villus length-to-crypt depth), were measured under 40× magnification.

### 5.5. Immunohistochemistry (IHC)

The paraffin sections (5 μm) were dewaxed and rehydrated regularly, and the antigen retrieved was used the microwaving method for about 20 min in 0.01-mol/L, pH 6.0 sodium citrate buffer. Subsequently, the sections were sealed with 3% H_2_O_2_ for about 30 min in order to block the endogenous peroxidase, then incubated in 10% normal calf or goat serum (ZSGB-BIO, Beijing, China) for about 30 min to block the nonspecific binding. Hereafter, we incubated the sections with mouse anti-PCNA monoclonal antibody (1:200, ZSGB-BIO) or rabbit anti-ghrelin polyclonal antibody (1:100, BIOSS, Beijing, China) at 4 °C overnight. On the following day, the sections were washed using 0.01-mol/L, pH 7.2 PBS and were subsequently covered using the corresponding secondary antibody for 1–1.5 h at 37 °C, according to the immunohistochemical enhanced kit instructions of the labeled polymer system kit (Polink-2 plus polymer HRP detection system for mouse or rabbit specific primary antibody, PV-9002/PV-9001, ZSGB-BIO); subsequently, the sections were washed with 0.01-mol/L, pH 7.2 PBS, followed by immersion in a DAB horseradish peroxidase color development kit (DAB kit, Tiangen for about 1–3 min to detect the result of immunohistological staining. At last, the slides were counterstained with hematoxylin dye and observed the distribution of immuno-positive cells with the yellow/brownish yellow color of the immunoreactive substances under a light microscope. At the same time, the negative control tissues sections were conducted with the same program, except that the specific primary antibody was substituted with PBS and 10% goat serum.

### 5.6. Measurement of the Integrated Optical Density

Ghrelin and PCNA labeling were examined by a microscope (Olympus BX41 (DP25), Japan). From each specimen, views of the fields in 10 stained sections were randomly chosen (*n* = 10 sections, per treatment) and photographed using the Olympus microscope camera system (Olympus BX41 (DP25), Japan). The next step was using Image Pro-Plus 6.0 analysis software (Media Cybernetics, MD, Maryland, USA) to analyze and evaluate the amount of immuno-positive cells stained. The SIOD (Integrated Optical Density of the single small intestinal villi) and IOD (Total Integrated Optical Density of a cross-section) were obtained, which were applied to compare the ghrelin and PCNA staining intensities in the duodenum, jejunum and ileum between the control and ZEA treatment.

### 5.7. Quantitative Real-Time PCR

Total RNAs were extracted from the small intestine samples with RNAiso Plus (Lot: AA4602-1, TaKaRa, Beijing, China), according to the kit manufacturer’s instructions and the literature of Dai et al. [24]. The concentration and purity of the RNA was evaluated using an Eppendorf biophotometer (Lot: RS323C, Eppendorf, Germany). The integrity of the total RNA was checked by AGE (agarose gel electrophoresis); then, we moved onto the next step, using the PrimeScript^TM^ RT Master Mix reverse transcription system kit (RR036A, TaKaRa, Beijing, China); the total RNA was reverse-transcribed onto the cDNA.

A total 20-μL volume of the qRT-PCR reaction mixture were contained with 6.8 μL of dH_2_O; 0.4 μL (10 μmol/L) of forward primers and 0.4 μL (10 μmol/L) of reverse primers, 10 μL of SYBRY Premix Ex Taq II (Lot: AK7502; TaKaRa, Beijing, China), 0.4 μL of ROX reference Dye II and 2.0 μL of the cDNA (<100 ng). The protocol of qRT-PCR consisted of an initial denaturation step at 95 °C for 30 s, followed by 40 cycles for 5 s at 95 °C, for 34 s at 60 °C, for 15 s at 95 °C and for 60 s at 60 °C; the final step was for 15 s at 95 °C. The reactions were conducted in the ABI 7500 Real-Time PCR System (Applied Biosystems, Foster City, CA, USA). The ghrelin and PCNA relative mRNA quantification levels were calculated and expressed as being the 2^−ΔΔCT^ [65] method. The qRT-PCR experiments were carried out in triplicate. In Table 4, the primer sequences of PCNA, ghrelin and GAPDH glyceraldehyde-3-phosphate dehydrogenase and the production lengths were presented.

### 5.8. Western Blot Analysis

The total protein of the small intestine was extracted using the Protein Extraction Kit (Beyotime, Shanghai, China) according to the kit manufacturer’s instructions, and the concentration was determined using the BCA protein quantitative kit (Tiangen). An equivalent amount of protein (40 μg) was loaded onto SDS-PAGE and blotted onto a PVDF (polyvinylidene difluoride) membrane. Then, the PVDF membranes were incubated with rabbit anti-ghrelin polyclonal antibody (1:250, BIOSS), mouse anti-PCNA monoclonal antibody (1:300, ZSGB-BIO) and mouse monoclonal anti-β-actin antibody (1:3000, Beyotime) at 4 °C for 12 h. After washing with pH 7.2, 0.01-mol/L TBST, the PVDF membranes were soaked with an anti-rabbit/mouse horseradish peroxidase-labeled antibody (1:3000) for 2.5 h at room temperature. The next step was incubating the PVDF membranes with a BeyoECL Plus kit (Beyotime), followed by detection with FusionCapt Advance FX7 (Beijing Oriental Science and Technology Development Co. Ltd., Beijing, China), and were quantified using Image Pro-Plus 6.0 software (Media Cybernetics, Sliver Springs, MD, USA).

### 5.9. Statistical Analysis

All data were expressed as the mean ± SD. In order to confirm the differences between the treatments, the one-way ANOVA and independent-sample *t*-test of the SPSS method for Windows (version 14.0) of the analyzed data were used, with *p* < 0.05 being considered a significant difference.

## Figures and Tables

**Table 1:** Zearalenone contents in the serum of weaned gilts (μg/mL).

Items	Zearalenone	α-Zearalenol	β-Zearalenol
Control	0.00 ± 0.00 ^b^	0.291 ± 0.02 ^b^	0.035 ± 0.01 ^b^
ZEA1.0	0.15 ± 0.04 ^a^	0.91 ± 0.10 ^a^	0.77 ± 0.01 ^a^

Data were presented by the mean ± SD (*n* = 6). ^a,b^ Means in the same column were significantly different (*p* < 0.05).

**Table 2:** Morphometric analysis of the small intestine in weaned gilts.

Items		Villus Length (μm)	Crypt Depth (μm)	VL/CD
Duodenum	Control	515.91 ± 7.43 ^a^	713.31 ± 7.58 ^b^	0.73 ± 0.01 ^a^
	ZEA1.0	355.86 ± 7.66 ^b^	880.36 ± 12.76 ^a^	0.41 ± 0.01 ^b^
Jejunum	Control	1059.37 ± 19.13 ^a^	493.21 ± 7.03 ^b^	2.16 ± 0.05 ^a^
	ZEA1.0	376.12 ± 13.49 ^b^	847.03±7.01 ^a^	0.59 ± 0.03 ^b^
Ileum	Control	334.67 ± 9.85 ^a^	895.51 ± 10.68 ^b^	0.48 ± 0.01 ^a^
	ZEA1.0	178.83 ± 8.68 ^b^	1759.83 ± 23.93 ^a^	0.10 ± 0.01 ^b^

Data were presented by the mean ± SD (*n* = 6). ^a,b^ Means in the same column were significantly different (*p* < 0.05). VL/CD, villus length/crypt depth.

**Table 3:** The compositions and ingredients of the basal diet.

Ingredients (%)	Content	Nutrients (%)	Analyzed Values
Corn	53.00	Metabolizable energy, MJ/kg	13.22
Whey powder	6.50	Crude protein	19.40
Wheat middling	5.00	L-Lysine HCl	0.30
Sodium chloride	0.20	Sulfur amino acid	0.79
Soybean oil	2.50	Total phosphorus	0.73
Limestone, Pulverized	0.30	Threonine	0.90
Fish meal	5.50	Methionine	0.46
Calcium phosphate	0.80	Tryptophan	0.25
Soybean meal	24.76	Lysine	1.36
L-threonine	0.04	DON mg/kg	0.41
DL-methionine	0.10	AFL ug/kg	1.62
Calcium	0.84	FUM mg/kg	0.28
Premix ^1^	1.00	ZEA mg/kg	0.14
Total	100		

^1^ Supplied per kg of diet: VE, 24 IU; VA, 3300 IU; K_3_, 0.75 mg; D_3_, 330 IU; B_12_, 0.02625 mg; B_6_, 2.25 mg; B_2_, 5.25 mg; B_1_, 1.50 mg; niacin, 22.5 mg; pantothenic acid, 15.00 mg; biotin, 0.075 mg; Mn (MnSO_4_·H_2_O), 6.00 mg; Zn (ZnSO_4_·H_2_O), 150 mg; Fe (FeSO_4_·H_2_O), 150 mg; Cu (CuSO_4_·5H_2_O), 9.00 mg; Se (Na_2_SeO_3_), 0.45 mg. folic acid, 0.45 mg; I (KIO_3_), 0.21 mg.

**Table 4:** Primers sequences of *GAPDH, ghrelin and* *PCNA*.

Target Genes	Accession No.	Primer Sequences	Product (bp)
*Ghrelin*	AF_308930	F: CCGAACACCAGAAAGTGCAG	144
		R: CGTTGAACCGGATTTCCAGC	
*PCNA*	NM_001291925.1	F: GTGATTCCACCACCATGTTC	145
		R: TGAGACGAGTCCATGCTCTG	
*GAPDH*	NM_001206359.1	F: ATGGTGAAGGTCGGAGTGAA	154
		R: CGTGGGTGGAATCATACTGG	

## Data Availability

Not applicable.
